# Direct Photocurrent Detection of Optical Vortex Based on the Orbital Photo Galvanic Effect: Progress, Challenge, and Perspective

**DOI:** 10.1002/advs.202519333

**Published:** 2026-04-07

**Authors:** Jinluo Cheng, Dehong Yang, Weiming Wang, Chang Xu, Zipu Fan, Dong Sun

**Affiliations:** ^1^ School of Physics and Laboratory of Zhongyuan Light Zhengzhou University Zhengzhou China; ^2^ GPL Photonics Laboratory State Key Laboratory of Luminescence Science and Technology Changchun Institute of Optics Fine Mechanics and Physics Chinese Academy of Sciences Changchun China; ^3^ University of Chinese Academy of Science Beijing China; ^4^ International Center for Quantum Materials School of Physics Peking University Beijing China; ^5^ Collaborative Innovation Center of Quantum Matter Beijing China; ^6^ Frontiers Science Center for Nano‐optoelectronics School of Physics Beijing China

**Keywords:** direct OAM detection, electric quadrupole and magnetic dipole interaction, orbital photo galvanic effects, photodetector

## Abstract

A photodetector that can directly distinguish the orbital angular momentum (OAM) of light is highly desirable for integrated on‐chip OAM detection and focal plane array devices. The recent development of OAM detectors based on the intrinsic orbital photo galvanic effects (OPGE) of materials provides a new route for direct OAM detection that is on‐chip scalable with high resolution and speed. In this paper, we summarize the current progress in direct photodetection of OAM via OPGE. We begin with a short review of the basic operation scheme of the OAM detector and provide a comprehensive symmetry analysis to sort out the favorable characteristics of the materials, incorporating considerations from device schemes based on various device performance characteristics and specific application circumstances. From that, we review the current experimental progress and technical challenges, then oversee the possible solutions to these challenges and provide a perspective on the future opportunities of this OAM detection route.

## Graphical abstract

Direct orbital angular momentum (OAM) detection through the orbital photogalvanic effect offers a scalable route for integrated optoelectronics. This perspective evaluates symmetry‐driven material selection and demonstrates how electrode matrices enable the resolution of complex, mixed OAM modes. By overcoming current technical challenges, these developments pave the way for high‐speed, on‐chip OAM sensing and focal plane arrays.

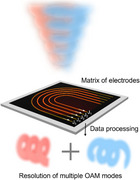

## 1 Introduction

**FIGURE 1:**
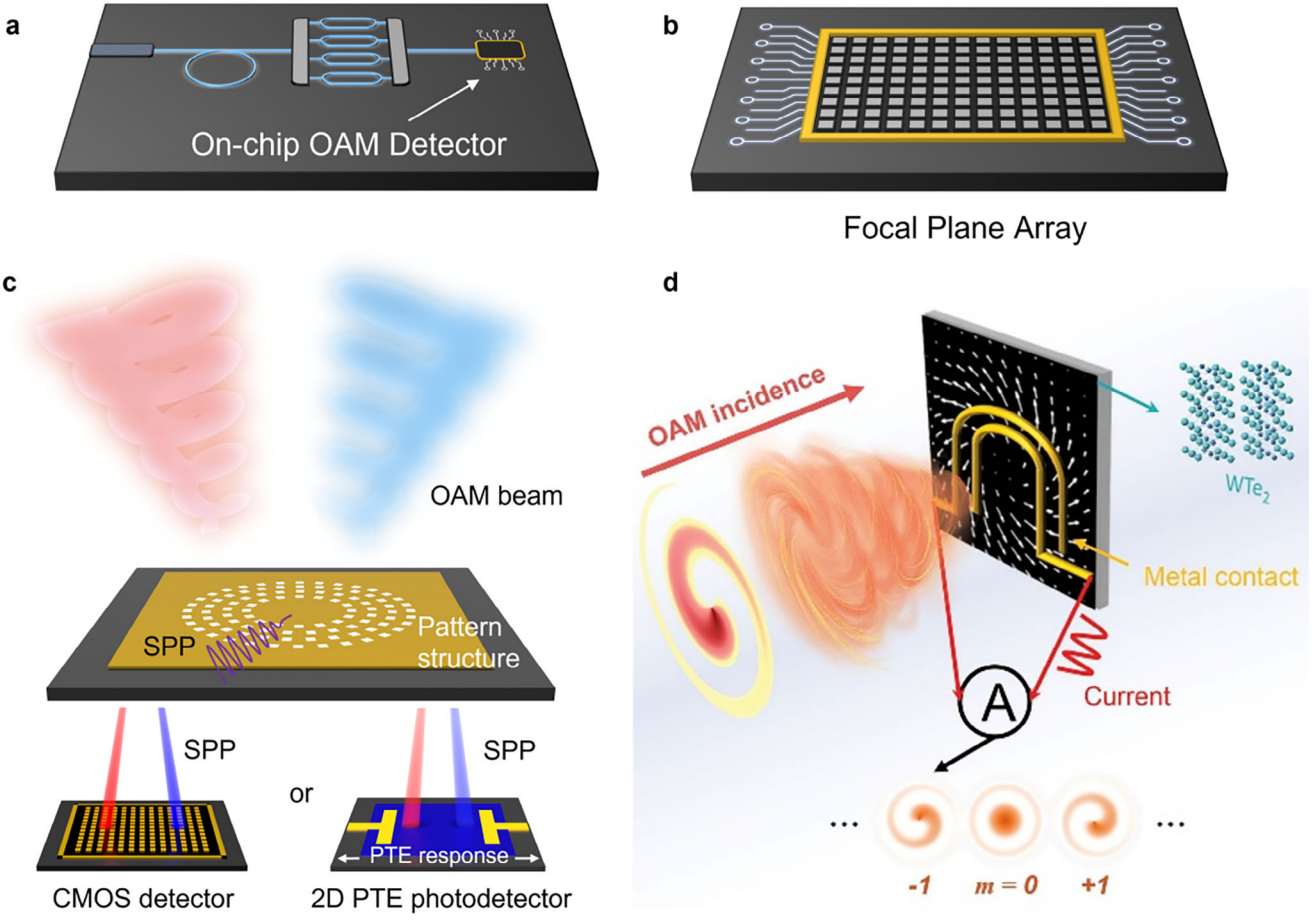
Schematic of two typical application perspectives and two typical technical pathways of direct OAM photodetection. (a,b) Schematic of the direct OAM detector used for on‐chip OAM circuits (a) and focal plane arrays (b). (c,d) Schematic of the SPP‐based (c) and OPGE‐based (d) OAM photodetection. Reproduced with permission [32], Copyright 2020, American Association for the Advancement of Science.

**FIGURE 2:**
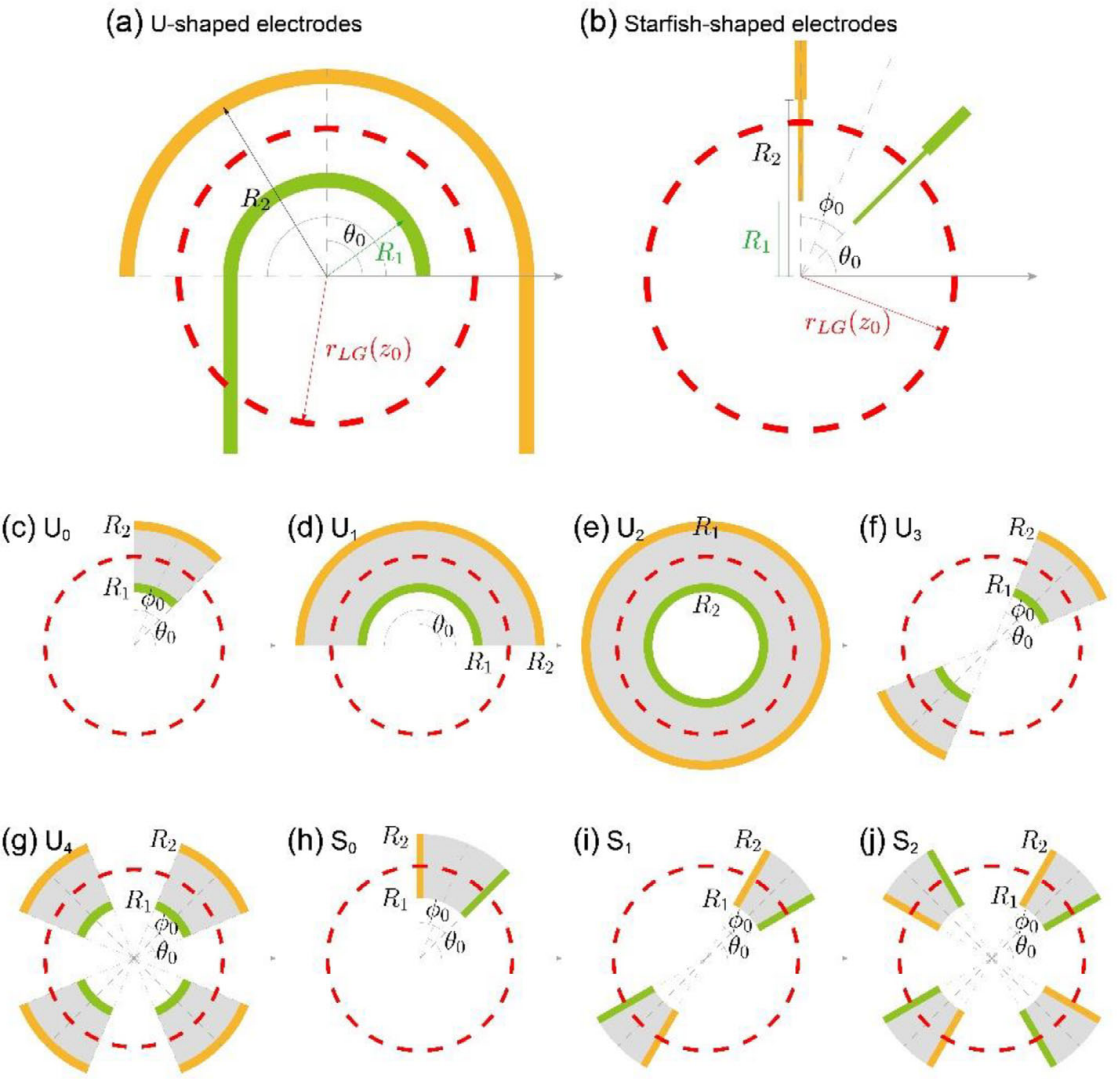
Schematic of different electrode designs. Illustration of the experimentally used U‐shaped electrodes (a) and starfish‐shaped electrodes (b). The LG beams with different OAM orders are focused to the same ring radius ***r***_***LG***_(***z***_0_), represented by the red dashed line. (c–j) Illustration of different types of electrodes. For each type of electrode, the yellow bars denote one end, and the green bars denote the other end of a pair of electrodes that are connected together, and the shadow region indicates the region where the current is collected from. (c‐j) Illustration of different types of electrodes used to collect radial currents (c‐g) and azimuthal currents (h‐j). (c) The U_0_ electrodes that collect current from the region formed by ***S*** ^***ρ***^(***θ***_0_,ϕ_0_), which is an intersection between a sector confined by two angles ***θ***_0_ ± ϕ_0_/2 and an annulus confined by two radii **R**_1_,**R**_2_. (d) The U_1_ electrodes that collect current from the region formed by **S** ^**ρ**^(***θ***_0_, ***π***). (e) The U_2_ electrodes that collect current from the region formed by **S** ^**ρ**^(***θ***_0_,2 ***π***). (f) The U_3_ electrodes that collect current from two regions formed by **S** ^**ρ**^(***θ***_0_,ϕ_0_) and **S** ^**ρ**^(***π*** + ***θ***_0_,ϕ_0_). (g) The U_4_ electrodes that collect current from four regions formed by **S** ^**ρ**^(***θ***_0_,ϕ_0_), $S^{\rho }\left(\frac{\pi }{2}+\theta_{0},\phi_{0}\right)$, **S** ^**ρ**^(***π*** + ***θ***_0_,ϕ_0_), and $S^{\rho }\left(\frac{3\pi }{2}+\theta_{0},\phi_{0}\right)$. (h) The **S**_0_ electrodes that collect current from the region formed by **S** ^**θ**^(***θ***_0_,ϕ_0_). (i) The **S**_1_ electrodes that collect current from the region formed by **S** ^***θ***^(***θ***_0_,ϕ_0_) and **S** ^**θ**^(***π*** + ***θ***_0_,ϕ_0_). (j) The **S**_2_ electrodes that collect current from the region formed by four regions: **S** ^***θ***^(***θ***_0_,ϕ_0_), $S^{\theta }\left(\frac{\pi }{2}+\theta_{0},\phi_{0}\right)$, **S** ^***θ***^(***π*** + ***θ***_0_,ϕ_0_), and $S^{\theta }\left(\frac{3\pi }{2}+\theta_{0},\phi_{0}\right)$.

Since the vortex solutions of the Maxwell‒Bloch equations were found and the concept of optical vortex (OVs) was introduced [1] in 1989, OVs technology has developed rapidly. Specifically, the orbital angular momentum (OAM) of light was experimentally discovered by Allen et al. in OVs with helical wavefronts [2], thereby opening a new area of research [3, 4, 5, 6]. OVs with OAM are widely used in various applications, ranging from optical manipulation [7, 8] to machining [9, 10], imaging [11, 12], optical communications [13, 14], quantum entanglement [15, 16], and even astronomical surveys [17, 18]. The generation [19, 20, 21, 22, 23], manipulation, and detection [24, 25, 26, 27, 28, 29] of OVs lay the foundation for various OAM‐based technologies. Among them, the detection technology of OAM is indispensable for any OAM‐related application. Conventional OAM measurement technologies usually rely on counting the stripes and lattices in the special interferogram and diffraction patterns to extract the phase information of OAM [27, 30], which requires bulky and complex setups, thereby imposing a fundamental limit on realizing a miniaturized, integrable, and fast operating detector of OAM. The recent rapid development of OAM light sources and manipulation at the nanoscale has enabled the on‐chip application of OAM, greatly promoting the demand for on‐chip photodetection of OAM [31], especially with direct electrical readout of OAM (Figure 1a). Equally important is the application circumstance whenever focal plane imaging of a target with OAM is needed (Figure 2b). In such applications, a large number of focal plane arrays of OAM detectors with electric readouts that are compatible with electric readout circuits are needed, with additional requirements for operation speed and unit cell size to achieve high‐resolution and high‐speed operation to acquire a clear image of a high‐speed target. However, such on‐chip electrical photodetection of OAM remains challenging with very limited prototype solutions.

To date, there are only two independent technical paths toward on‐chip electrical photodetection of OAM. The first pathway is based on surface plasmon polaritons (SPPs), which relies on well‐designed plasmonic nanostructures to separate various OAM modes into focused SPPs at different spatial positions [33, 34, 35, 36, 37, 38]. Then, different OAM modes are resolved by detecting the SPPs. The detection usually requires far‐field detectors to characterize the transferred SPPs or a near‐field scanning microscope to record the focused SPPs, which limits system‐level integration. Several different schemes have been proposed to solve this issue. For example, by integrating a holographic coupler with a commercial photodiode, certain OAM modes can be detected directly, but others cannot be effectively distinguished [35]. More recent work has used a spin‒Hall SPP coupler to sort out different OAM modes and then detect them via the thermoelectric response of PdSe_2_, which can distinguish different OAM modes simultaneously and demonstrate direct on‐chip detection of spin and orbital angular momentum, as well as the chirality and ellipticity of scalar vortex light (Figure 1c) [39]. The second pathway is based on the orbital photogalvanic effect (OPGE) of selected materials, which is the focus of this paper. This orbital photogalvanic effect is driven by the helical phase gradient of light OAM through the electric quadrupole and magnetic dipole response of materials, and then the OAM‐dependent photocurrent response component can be extracted from the measurement of the circular polarization‐dependent photogalvanic response. The magnitude of the extracted OPGE is proportional to the quantized OAM mode number, which resolves the OAM order of the incident light. Such a scheme was first proposed and realized in the near‐infrared wavelength range by Ji et al. using the type‐II Weyl semimetal WTe_2_ in 2020 (Figure 1d) [32]. After this conceptual demonstration, the recent developments have expanded the functional wavelength range and significantly increased the operation speed [40, 41]. Moreover, such a detection scheme was recently found to be not limited to Weyl semimetals; a wide range of materials also work similarly with pronounced OPGE responses, even in the challenging mid‐infrared wavelength range [42]. A comparison of these detection methods is listed in Table 1.

**TABLE 1:** A comparison of different OAM detection methods.

	Traditional methods [27, 30]	SPP‐based methods		OPGE‐based method [42]
		Near field image [35]	Spin‐Hall SPP coupler [39]	
Working principle	interferogram and diffraction patterns	well‐designed metasurfaces to convert OAM modes into focused SPPs at different spatial resolutions	Detecting the focused SPP using the thermoelectric response of PdSe_2_	specially designed electrodes to collect the OAM order quantized photocurrent
Wavelength	flexible	633 nm	8 µm	4 µm
Device footprint	bulky	A few hundred µm	200 × 200 µm	40 × 40 µm
Operation Speed	very slow	depending on the detector	69/25 µs	1 ms
OAM order	flexible	specific	[−4, 4]	[−4, 4]
Responsivity	/	tens of µA/W	0.2 nA/W	151.4 nA/W
System‐level integration	N	Y	Y	Y

With recent rapid developments, such a direct OAM detection scheme based on OPGE promises unprecedented opportunities for further development toward scalable, high‐resolution, high‐speed, on‐chip direct OAM photodetectors. In this work, we provide a perspective on the future development opportunities of this technology pathway based on a comprehensive overview of the current technical challenges, and oversee the possible solutions to these challenges and opportunities in the future. We start with a short review of the basic operation scheme of the detector based on OPGE and recent experimental progress. Then the paper features a comprehensive symmetry analysis of all crystal symmetries and then sorts out the favorable characteristics of the material candidates from a symmetry point of view while incorporating the consideration of device scheme based on various device performance characteristics and specific application circumstances, which has not been published elsewhere yet. Based on these results, we discuss in detail how the symmetry of the electrodes affects the characterization of the OAM response current as well as the signal resolution; then, we review the experimental progress on various devices made from different functional materials and the recent progress on improving the operation speed using photoelastic modulator. Furthermore, a new OAM detection strategy by adjusting the linear polarization of the LG beams is proposed. Finally, we discuss the challenge and perspective on the direct detection of OAM order.

## 2 OAM Detection via OPGE: Operation Mechanism, Device Scheme, and Experimental Progress

In this section, we first show theoretically how the OAM of light interacts with OAM‐sensitive materials and contributes to an OAM‐dependent photocurrent response, known as OPGE. Furthermore, we discuss the typical device scheme and operation mechanism based on OPGE.

### 2.1 Operation Mechanism

Though the structured light can have significant effects on the photocurrent due to the peculiar spatial distribution of the light intensity [43, 44], the direct detection of light OAM through the photocurrent response is usually difficult because the photocurrent response does not inherently carry phase information of the incident light, while the OAM of light reflects a peculiar phase variation in the electric field. Except in special nanostructures [43, 44] or experimental conditions [45], the light OAM degree of freedom is difficult to interact with electron's “internal” degree of freedom [46, 47], e.g., the band index in a crystal, because of the huge scale mismatch between the micrometer scale of light OAM and the nanometer scale of electron's wave function; thus, the electron state can only see the local field of the structured light, instead of its global phase properties. In the approximation of electric dipole interactions, the photocurrent response corresponds to the conventional second‐order photogalvanic effect, which is determined by the local intensity and polarization of light, irrelevant to the OAM order. The photocurrent response to light OAM can emerge from the phase gradient of optical fields through interactions with the electric quadrupole and magnetic dipole. The spatial variation of OAM light is on the order of wavelength (∼ µm), which is usually much larger than the scale of the unit cell (∼Å). The several‐order length scale differences limit the microscopic interaction between the OAM and solid materials. Theoretically, for a light field ***E*** (***r***, *t*) = ***E*** (***r***, ω)*e* ^−*i*ω*t*^ + *c*.*c*. interacting with materials, the spatial distribution of the generated DC current can be described as:

(1) jardc=2ReαabcωEbr,ωEc∗r,ω+4Imβabcdω∂Ebr,ω∂rdEc∗r,ω

where the subscripts *a*, *b*, *c*, *d* denote the directions in the Cartesian coordinate system; the response coefficient α_*abc*_(ω) is a third‐rank tensor, which describes the conventional second‐order response resulting from the dipole approximation, and the response coefficient β_*abcd*_(ω) is a fourth‐rank tensor, which describes the effects from the gradient of the electric field through the electric quadrupole and magnetic dipole interaction. The response coefficients α_*abc*_(ω) and β_*abcd*_(ω) are connected to the general second‐order optical conductivities σ_*abc*_(***q***, ω; ***q′***, −ω) in the long wavelength limit ***q***, ***q′*** ∼ 0 as:

(2) $$\sigma_{abc}\;\left(q,\omega ;q^{\prime },-\omega \right)=\alpha_{abc}\left(\omega \right)+q_{d}\beta_{abcd}\left(\omega \right)+q_{d}^{\prime }\beta_{acbd}\left(-\omega \right)$$

The β_*abcd*_ term in Equation (1) describes a part of the spatially nonlocal current that is related to the OAM of light. To elaborate on the effect of the helical phase gradient of light OAM in the response, we write the profile of the light field carrying OAM propagating along the *z* direction in the cylindrical coordinate system:

(3) $$\begin{array}{c} E\;\left(r,\omega \right)=E_{0}\;u_{p,\left|m\right|}\left(\rho ,z\right)e^{im\theta }\frac{\hat{x}+\sigma \hat{y}}{\sqrt{1+{\left|\sigma \right|}^{2}}} \end{array}$$

with σ = σ_*r*_+ *i*σ_*i*_ describing the light elliptical polarization, $r\;=\rho \cos\theta \;\hat{x}+\rho \sin\theta \;\hat{y}+z\;\hat{z}$ and

(4) $$\begin{array}{ccc} u_{p,\left|m\right|}\;\left(\rho ,z\right) & = & C_{pm}\frac{\;w_{0}}{w\left(z\right)}\;{\left(\frac{\sqrt{2}\rho }{w\left(z\right)}\right)}^{\left|m\right|}L_{p}^{\left|m\right|}\left(\frac{2\rho^{2}}{w^{2}\left(z\right)}\right)\\ & & \times \;\exp\left[-\frac{\rho^{2}}{w^{2}\left(z\right)}+i\left(2p+\left|m\right|+1\right)\eta \left(z\right)-i\frac{k\rho^{2}}{2q\left(z\right)}\right] \end{array}$$

where E_0_ is the electric field amplitude, $C_{pm}=\sqrt{2p!/\pi (p+|m|)!}\;\;$ is a normalization coefficient, *w*_0_ is the waist‐spot radius of the Gaussian beam, $w\left(z\right)=w_{0}\;\sqrt{1+z^{2}/z_{0}^{2}}$ is the spot radius at position *z*, *m* is the angular quantum number or OAM order number, *p* is the radial quantum number, $L_{p}^{m}\;\left(x\right)=\frac{x^{-m}e^{x}}{p!}\;\frac{d^{p}}{dx^{p}}\left(e^{-x}x^{p+m}\right)$ is the generalized Laguerre polynomial, $z_{0}=\pi w_{0}^{2}/\lambda$ is the Rayleigh length, $\eta \left(z\right)=\arctan\left(z/z_{0}\right)$ is the Gouy phase, and $q\;\left(z\right)=z+z_{0}^{2}/z$ is the radius of curvature. Owing to the spatial derivative in Equation (1), the OAM order *m* appears in the response for light field carrying OAM, providing an OAM‐dependent photo galvanic response. The spatial distribution of the photocurrent ***j*** ^dc^(***r***) generated by the light field in Equation (3) can be sorted according to the parity dependence of *m* and σ as:

(5) $$\begin{array}{ccc} ja_{\left(r\right)}^{\mathrm{dc}} & = & m\left[\sigma_{i}j_{a}^{\left(1\right)}\left(r\right)+\sigma_{r}j_{a}^{\left(5\right)}\left(r\right)\right]\\ & & +\;mj_{a}^{\left(2\right)}\left(r\right)+\left[\sigma_{i}j_{a}^{\left(3\right)}\left(r\right)+\sigma_{r}j_{a}^{\left(6\right)}\left(r\right)\right]+j_{a}^{\left(4\right)}\left(r\right)\; \end{array}$$

where $j_{a}^{\left(n\right)}\left(r\right)$ with *n*  =  1, 2, ···, 6 are given as:

(6) ja1=Imβaxyy−βayxyf1g1+βayxx−βaxyxf1g2,ja2=Reβaxxy+σ2βayyyf1g1−βaxxx+σ2βayyxf1g2,ja3=Imαaxy−αayxf0g0+Reβayxx−βaxyxf2g1−βaxyy−βayxyf2g2,ja4=Reαaxy+αayxf0g0+Imβaxxx+σ2βayyxf2g1+βaxxy+σ2βayyyf2g2,ja5=Reβaxyy+βayxyf1g1−βaxyx+βayxxf1g2,ja6=Reαaxx+σ2αayyf0g0+Imβaxyx+βayxxf2g1+βaxyy+βayxyf2g2.

In Equation (6), the position dependences of $j_{a}^{\left(n\right)}\left(r\right)$, *f_j_* (ρ,*z*), and *g_j_* (θ) with *j*  =  0, 1, 2 are not shown explicitly; the functions *f_j_* and *g_j_* depend solely on the radial coordinate ρ and the azimuthal angle θ separately, and they can be written as:

(7) $$\begin{array}{ccc} f_{0}\left(\rho ,z\right) & = & \frac{2{\left|E_{0}\right|}^{2}{\left|u_{p,\left|m\right|}\left(\rho ,z\right)\right|}^{2}}{1+{\left|\sigma \right|}^{2}},\;f_{1}\left(\rho ,z\right)=\frac{2}{\rho }f_{0}\left(\rho ,z\right)\;,\\ f_{2}\left(\rho ,z\right) & = & \frac{2f_{0}\left(\rho ,z\right)}{u_{p,\left|m\right|}\left(\rho ,z\right)}\frac{\partial u_{p,\left|m\right|}\left(\rho ,z\right)}{\partial \rho },\;g_{0}\left(\theta \right)=1\;,\\ g_{1}\left(\theta \right) & = & \cos\theta ,\;g_{2}\left(\theta \right)=\sin\theta \;.\; \end{array}$$

The current distribution $j_{a}^{\mathrm{dc}}\left(r\right)$ in Equation (5) has the following features:

(1) All terms of $j_{a}^{\left(n\right)}$ depend on σ through the factor 1/(1 + |σ|^2^) and show a complicated dependence on m through functions *f*_0_, *f*_1_, and *f*_2_; however, all of them are even functions of *m* and σ, which can be observed from Equations (6 and 7).

(2) The photocurrent distribution $j_{a}^{\mathrm{dc}}\left(r\right)$ in Equation (5) is divided into six terms, each of which is an even or odd function of the OAM order *m* and the elliptical polarization σ: the term $m\sigma_{i}j_{a}^{\left(1\right)}\left(r\right)$ is an odd function of both *m* and σ_*i*_ but an even function of σ_r_; thus, the current direction from this term reverses as one of *m* and σ_*i*_ switches signs; the term $m\sigma_{r}j_{a}^{\left(5\right)}\left(r\right)$ is an odd function of both *m* and σ_*r*_ but an even function of σ_*i*_; the term $mj_{a}^{\left(2\right)}\left(r\right)$ is an odd function of *m* but an even function of both *m*, σ_*r*_ and the term σ_*i*_; the term $\sigma_{i}j_{a}^{\left(3\right)}\left(r\right)$, is an odd function of σ_*i*_ but an even function of both *m* and σ_*r*_; the term $\sigma_{r}j_{a}^{\left(6\right)}\left(r\right)$ is an odd function of σ_*r*_ but an even function of both m and σ_*i*_; and the term $j_{a}^{\left(4\right)}\left(r\right)$ is an even function of *m*,  σ_*r*_, σ_*i*_.

(3) The signs of *m*, σ_*r*_ and σ_*i*_ are experimentally controllable, and the contributions of these six terms to $j_{a}^{\mathrm{dc}}\left(r\right)$ can be distinguished via a series of experiments by changing the signs of *m*, σ_*r*_ and σ_*i*_ only. For example, the CPGE measurement provides the difference in the photocurrent responses between left circularly polarized light (σ_*r*_ =  0,  σ_*i*_ = +1) and right circularly polarized light (σ_*r*_ =  0,  σ_*i*_ = −1), which is contributed by the terms $mj_{a}^{\left(1\right)}\left(r\right)+j_{a}^{\left(3\right)}\left(r\right)$; alternatively, the usual OPGE measurement provides the difference between the CPGE current for OAM order m and OAM order −m, which is contributed by the term $mj_{a}^{\left(1\right)}\left(r\right)$.

According to Equations (5) and (6), The minimum requirement for a certain material to have a possible OPGE response is to have nonvanishing coefficients of β_*axyy*_ − β_*ayxy*_ or β_*ayxx*_ − β_*axyx*_ for *a*  =  *x*, *y*. In fact, such conditions can be easily fulfilled for any crystal because, from the symmetry point of view, the tensor components. β_*xxyy*_, β_*xyxy*_, β_*yyxx*_, and β_*yxyx*_ are always nonzero for any point group, and the spatial distribution of $j_{a}^{\mathrm{dc}}\left(r\right)$ does not vanish. However, on one hand, the symmetry tells whether there are nonvanishing. β_*abcd*_ terms or OPGE terms, it does not tell the response amplitude; on the other hand, there are additional limits imposed from practical considerations, such as device structures and background noise. If the electrodes are considered further, for metallic materials, the current *I*(*m*, σ) collected by a certain electrode geometry according to the Shockley ‒Ramo theorem [48, 49, 50] can be written as:

(8) $$\begin{array}{ccc} I\left(m,\sigma \right) & = & \int j^{\mathrm{dc}}\left(r\right)\cdot e\left(r\right)dr=m\sigma_{i}I^{\left(1\right)}+mI^{\left(2\right)}+\sigma_{i}I^{\left(3\right)}+I^{\left(4\right)}\\ & & +\;m\sigma_{r}I^{\left(5\right)}+\sigma_{r}I^{\left(6\right)}\;\\ I^{\left(n\right)} & = & \int j^{\left(n\right)}\left(r\right)\cdot e\left(r\right)dr\;\; \end{array}$$

where ***e*** (***r***) is an auxiliary weighting field determined by solving Laplace's equation within a specific device geometry, and in nonmagnetic materials, it is proportional to the electric field distribution when a voltage is applied to the electrodes. In Equation (8), the expressions of *I* ^(*n*)^ can be written as functions of the coefficients α_*abc*_ and β_*abcd*_ as:

(9) I1=∑aImβaxyy−βayxyF11a+βayxx−βaxyxF12a,I2=∑aReβaxxy+σ2βayyyF11a−βaxxx+σ2βayyxF12a,I3=∑aImαaxy−αayxF00a+Reβayxx−βaxyxF21a−βaxyy−βayxyF22a,I4=∑aReαaxy+αayxF00a+Imβaxxx+σ2βayyxF21a+βaxxy+σ2βayyyF22a,I5=∑aReβaxyy+βayxyF11a−βaxyx+βayxxF12a,I6=∑aReαaxx+σ2αayyF00a+Imβaxyx+βayxxF21a+βaxyy+βayxyF22a.

with

(10) $$F_{jla}=\int f_{j}\left(\rho ,z\right)g_{l}\left(\theta \right)e_{a}\left(r\right)dr$$

A successful OAM device requires that there exist nonzero components of *F*_1*la*_ for *l*  =  1, 2 and *a*  =  *x*, *y*. Besides *f_j_* and *g_l_*, *F*_1*la*_ is also affected by the auxiliary weighting field ***e*** (***r***), which is related to the geometry of the electrodes, so *F*_1*la*_ needs to be optimized for better collection performance and favorable OPGE responses. Not all electrodes can provide nonzero values of *F_jla_*. A counterexample is the conventional two‐terminal rectangular electrode geometry, with ***e*** (***r***) being a constant vector, the value of *F*_1*la*_ is zero because the angle integrations of *g*_1_(θ) = cos θ  and *g*_2_(θ) = sin θ  are zero; thus, no OPGE current can be observed, which magnifies the critical role of the geometry of the electrodes, as discussed in the next section. In principle, all *F_jla_* values are functions of the OAM order *m*; however, for a suitable electrode geometry and spot size of the LG beam, all *F_jla_* values are very weakly dependent on *m* ^40^. This property is very important for accurately extracting different OAM orders in experiments, and it makes the measured current signal *I*(*m*, σ) shows a simple linear dependence on *m*. For example, there are three terms, *m*σ_*i*_ *I* ^(1)^, *mI* ^(2)^, and *m*σ_*r*_ *I* ^(5)^, linearly dependent on *m*. As long as any of the terms *I* ^(1)^, *I* ^(2)^, or *I* ^(5)^ are nonzero, the photocurrent signal *I*(*m*, σ) should show a linear dependence on *m*, which can be used to detect the OAM order. In all experimental works demonstrated so far, the OPGE signal is extracted from a CPGE measurement. Under this scheme, the current difference between opposite circularly polarized (σ = ±*i*) LG beams is measured, and the result is given by:

(11) $$\begin{array}{cc} I^{\mathrm{CPGE}}\left(m\right) & =\frac{1}{2}\left[I\left(m,i\right)-I\left(m,-i\right)\right]=mI^{\left(1\right)}+I^{\left(3\right)} \end{array}\;$$

Furthermore, the usual OPGE current can be separated by the term *mI* ^(1)^:

(12) $$\begin{array}{cc} I^{\mathrm{OPGE}} & =\frac{1}{2}\left[I^{\mathrm{CPGE}}\left(m\right)-I^{\mathrm{CPGE}}\left(-m\right)\right]=mI^{\left(1\right)} \end{array}\;$$

which is proportional to *m*. Therefore, it is *mI* ^(1)^ that dominates the CPGE response, and it is used for the OAM detection scheme based on CPGE extraction, and all other currents *I* ^(*n*)^ for *n*  =  2, 3, ···, 6 behave as background currents. As the OPGE current is extracted from the CPGE current, *I* ^(3)^ is the main background current, which should be minimized to reduce its interference to the measurement of *mI* ^(1)^. Now, the possibility of detection of the OAM order through CPGE extraction is converted to a problem of determining the situation in which the value of *I* ^(1)^ is nonzero while minimizing the background from other terms at the same time. In principle, the currents from the terms *mI* ^(2)^ and *m*σ_*r*_ *I* ^(5)^ can also be treated as the signal for OAM detection, but they have to be extracted by changing the degree of linear polarization, which remains an interesting opportunity to explore experimentally.

To date, the OPGE response and related direct OAM detection device have been experimentally demonstrated on three different materials: WTe_2_, TaIrTe_4_, and multilayer graphene (MLG) [32, 40, 42]. Among them, WTe_2_ and TaIrTe_4_ both belong to the C_2v_ point group and share the same symmetry analysis, while MLG belongs to the D_6h_ point group. In either case, the electrical dipole response, which is contributed by the third‐rank tensor α_*abc*_, vanishes under normal incidence conditions due to the lack of planar components α_*abc*_ for C_2v_ and the existence of inversion symmetry of D_6h_. For C_2v_, there are 21 independent, nonzero fourth‐rank tensors β_*abcd*_, including eight planar components (β_*xxxx*_, β_*xxyy*_,β_*xyxy*_, β_*xyyx*_ and β_*yyyy*_, β_*yyxx*_, β_*yxyx*_, β_*yxxy*_) and 13 nonplanar components (β_*zzzz*_, β_*zzxx*_, β_*zxzx*_, β_*zxxz*_, β_*xxzz*_, β_*xzxz*_, β_*xzzx*_, β_*zzyy*_, β_*zyzy*_, β_*zyyz*_, and β_*yyzz*_, β_*yzyz*_, β_*yzzy*_). With such symmetry, devices based on these WTe_2_ and TaIrTe_4_ materials are OAM sensitive for both the U‐shaped electrodes and starfish‐shaped electrodes [32, 40, 42]. For D_6h_, the 21 nonzero tensor components are the same as those of C_2v_, but only 10 of them are independent (see Table 3). With such relationships between different tensor components, devices based on MLG and starfish‐shaped electrodes become OAM‐insensitive. Therefore, a full symmetry analysis is necessary for the realization of direct OAM detection. Based on Equation (9), the detected OPGE current signals are determined jointly by the effects of three factors: (i) the geometry of the electrodes appearing in *F_jla_*, (ii) the response coefficients α_*abc*_ and β_*abcd*_, which are determined by the electronic properties of the functional material, and (iii) the polarization of the incident LG beams (*m*, σ). Here, the elliptical polarization σ in factor (iii) is treated as an adjustable parameter for detecting *m*, and factors (i) and (ii) will be discussed in sequence as follows.

### 2.2 Symmetry Analysis for the Geometry of Electrodes

Importantly, the peculiarly designed electrodes play a key role in the collection of OAM‐sensitive currents based on the idea that the generated photocurrents in the whole laser spot are collected with unequal weight. For the OPGE detectors that have already been demonstrated experimentally, the electrodes are usually designed into two types: (1) U‐shaped electrodes, as shown in Figure 2a, for collecting the photocurrent along the radial direction in the shadow region, and (2) Starfish‐shaped electrodes, as shown in Figure 2b, for collecting the photocurrent along the azimuthal direction in the shadow region. The shape and geometry of the electrodes affect the quantity *F_jla_* according to Equation (10), which depends on the auxiliary weight field ***e*** (***r***). For both types of electrodes, ***e*** (***r***) is distributed mainly in the shadow region from where the photocurrent is collected. This region can generally be represented as an intersection *S*(θ_0_, ϕ_0_) between a sector confined by two angles $\theta_{0}\pm \frac{\phi_{0}}{2}$ and an annulus confined by two radii *R*_1_, *R*_2_. From Figure 2a,b, the U‐shaped electrodes collect the photocurrent from the region formed by *S*(θ_0_, π) = *S* ^ρ^(θ_0_, π) with $e\;\left(r\right)=e^{\rho }\;\left(r\right)\equiv \;\cos\theta \;\hat{x}+\sin\theta \;\hat{y},$ and the starfish‐shaped electrodes collect the photocurrent from the region formed by *S*(θ_0_, ϕ_0_) = *S* ^θ^(θ_0_, ϕ_0_) and $e\;\left(r\right)=e^{\theta }\;\left(r\right)\equiv \;-\sin\theta \;\hat{x}+\cos\theta \;\hat{y}$; here, we use the superscript ρ, θ to indicate the two types of electrodes. The factor *F_jla_* in Equation (10) can be written as $F_{jla}=P_{j}\;G_{la}^{\rho /\theta }$, where

(13) $$\begin{array}{c} P_{j}=\int \int_{R_{1}}^{R_{2}}\rho f_{j}\left(\rho ,z\right)d\rho ,\;\;G_{la}^{\frac{\rho }{\theta }}=\int_{\theta_{0}-\frac{\phi_{0}}{2}}^{\theta_{0}+\frac{\phi_{0}}{2}}d\;\theta g_{l}\left(\theta \right){\hat{e}}_{a}^{\frac{\rho }{\theta }}\left(r\right)d\theta \end{array}$$

Here, ${\hat{e}}_{a}^{\rho }\left(r\right)$ (or ${\hat{e}}_{a}^{\theta }\left(r\right)$) for *a*  =  *x*, *y* is the *a*th component of the direction of the auxiliary weight field ***e*** ^ρ^(***r***) (or ***e*** ^θ^(***r***)). If the values of *F*_1*la*_ are all zero, the value of *I* ^(1)^ becomes zero, and the device becomes OAM‐insensitive regardless of the specific symmetry of the functional material used. In fact, this is indeed the case for conventional two‐terminal rectangular electrodes. Thus, it is important to analyze *F_jla_* for electrodes with different shapes and symmetries. In general, *P_j_* for *j*  =  0, 1, 2 depends on the radial profile of the LG beam and is not zero, whereas *G_la_* for *l*  =  0, 1, 2 and *a*  =  *x*, *y* depends on the geometry parameters θ_0_ and ϕ_0_, which can be strongly affected by the electrodes. The U‐shaped and starfish‐shaped electrodes used in the experiments have very low symmetry. In order to understand whether electrode symmetry can affect the OPGE current, more types of electrodes with higher symmetries are designed, as shown in Figure 2c–j, for collecting the radial and azimuthal currents. In Table 2, we list the values of *G_la_* for different types of electrodes, and below, we present a detailed discussion of how electrode symmetry affects the dependence of the photocurrent on the material's response coefficients:

**TABLE 2:** The factors ***G***_***la***_ with ***l *** = * *0, 1, 2 and ***a *** = *** x***, ***y*** for different types of electrodes listed in Figure 2.

(ê(r)r)	Type	Collection region	$\left(\begin{array}{c} G_{0x}\\ G_{0y} \end{array}\right)$	$\left(\begin{array}{c} G_{1x}\\ G_{1y} \end{array}\right)$	G2xG2yG2xG2y11
radial $\left(\begin{array}{c} \cos\theta \\ \sin\theta \end{array}\right)$	U_0_	*S* ^ρ^(θ_0_,ϕ_0_)	$2\sin\frac{\phi_{0}}{2}\left(\begin{array}{c} \cos\theta_{0}\\ \sin\theta_{0} \end{array}\right)$	$\left(\begin{array}{c} c_{1}\\ c_{2} \end{array}\right)$	c2c3G2xG2y11
	U_1_	*S* ^ρ^(θ_0_,π)	$2\left(\begin{array}{c} \cos\theta_{0}\\ \sin\theta_{0} \end{array}\right)$	$\left(\begin{array}{c} \frac{\pi }{2}\\ 0 \end{array}\right)$	0π2G2xG2y11
	U_2_	*S* ^ρ^(θ_0_,2π)	$\left(\begin{array}{c} 0\\ 0 \end{array}\right)$	$\left(\begin{array}{c} \pi \\ 0 \end{array}\right)$	0πG2xG2y11
	U_3_	$\sum_{l=0}^{1}S^{\rho }\left(l\pi +\theta_{0},\phi_{0}\right)$	$\left(\begin{array}{c} 0\\ 0 \end{array}\right)$	$\left(\begin{array}{c} 2c_{1}\\ 2c_{2} \end{array}\right)$	2c22c3G2xG2y11
	U_4_	$\sum_{l=0}^{3}S^{\rho }\left(\frac{l\pi }{2}+\theta_{0},\phi_{0}\right)$	$\left(\begin{array}{c} 0\\ 0 \end{array}\right)$	$\left(\begin{array}{c} 2\phi_{0}\\ 0 \end{array}\right)$	02ϕ0G2xG2y11
azimuthal $\left(\begin{array}{c} -\sin\theta \\ \cos\theta \end{array}\right)$	S_0_	*S* ^θ^(θ_0_,ϕ_0_)	$2\sin\frac{\phi_{0}}{2}\left(\begin{array}{c} -\sin\theta_{0}\\ \cos\theta_{0} \end{array}\right)$	$\left(\begin{array}{c} -c_{2}\\ c_{1} \end{array}\right)$	−c3c2G2xG2y11
	S_1_	$\sum_{l=0}^{1}S^{\theta }\left(l\pi +\theta_{0},\phi_{0}\right)$	$\left(\begin{array}{c} 0\\ 0 \end{array}\right)$	$\left(\begin{array}{c} -2c_{2}\\ 2c_{1} \end{array}\right)$	−2c32c2G2xG2y11
	S_2_	$\sum_{l=0}^{3}S^{\theta }\left(\theta_{0}+\frac{l\pi }{2},\phi_{0}\right)$	$\left(\begin{array}{c} 0\\ 0 \end{array}\right)$	$\left(\begin{array}{c} 0\\ 2\phi_{0} \end{array}\right)$	−2ϕ00G2xG2y11
c1=12(ϕ0+cos2θ0sinϕ0),c2=12sin2θ0sinϕ0,c3=12(ϕ0−cos2θ0sinϕ0)G2xG2y11					

(1) For the electrodes *U*_0_ in Figure 2c, the radial photocurrent is collected in the region S^ρ^(θ_0_,ϕ_0_). When θ_0_ and ϕ_0_ take general values, the electrodes do not possess any symmetry, and $G_{la}^{\rho }$ are all nonzero. In this case, whether the device is OAM sensitive is fully determined by the properties of α_abc_ and β_abcd_, and the OAM‐sensitive current *I* ^(1)^ depends on four material parameters: β_*axyy*_ − β_*ayxy*_ and β_*ayxx*_ − β_*axyx*_ with *a = x,y*. When the electrodes are placed at certain angles, for example, θ_0_ =  0, $G_{0y}^{\rho }=0$ and $G_{1y}^{\rho }=G_{2x}^{\rho }=0$, some of the tensor components appear in Equation (9), α_*abc*_ and β_*abcd*_, will no longer affect the values of *I* ^(*n*)^. Taking the OAM‐sensitive current *I* ^(1)^ as an example, it depends on the two material parameters β_*xxyy*_ − β_*xyxy*_ and β_*yyxx*_ − β_*yxyx*_ and not on β_*yxyy*_ − β_*yyxy*_ and β_*xyxx*_ − β_*xxyx*_. Therefore, the orientation of the electrodes *U*_0_ can effectively change the dependence of the OPGE current on the response coefficients.

For the experimentally used U‐shaped electrodes in Figure 2a, which are equivalent to the electrodes U_1_ in Figure 2d, they collect the radial photocurrent in the region *S* ^ρ^(θ_0_,π). This type of electrodes has mirror symmetry along the direction. θ_0_, and the coefficients $G_{la}^{\rho }$ satisfy $G_{1x}^{\rho }=G_{2y}^{\rho }=\pi /2$ and $G_{1y}^{\rho }=G_{2x}^{\rho }=0$; in this case, the current *I* ^(*n*)^ depends on many fewer material parameters because of the mirror symmetry: *I* ^(1)^ only depends on one parameter Im[β_*xxyy*_ − β_*xyxy*_ + β_*yyxx*_ − β_*yxyx*_], and *I* ^(3)^ depends on α_*axy*_ − α_*ayx*_ and β_*xyxx*_ − β_*xxyx*_ − β_*yxyy*_ + β_*yyxy*_. The advantages of such symmetry analysis can be seen as follows: in order to suppress the CPGE background current *I* ^(3)^, the inversion symmetric materials in class E_2_ (in Table 3) are also candidates for electrodes U_1_ for OAM detection; while for electrodes U_0_, such materials do not work, as will be extensively discussed later in the section “Crystal symmetry analysis for photocurrents *I* ^(*n*)^ with *n*  =  1, 2, ···, 6”. Therefore, analyzing the symmetry of electrodes is indispensable for selecting functional materials.

**TABLE 3:** Crystal symmetry analysis for the planar components of the response tensors α_*abc*_ and β_*abcd*_. The crystal system is adopted from Boyd's book [51], and the column “nonvanishing currents” denotes nonzero terms of *I* ^(*n*)^ for n  =  1, 2, ···, 6 for several types of electrodes listed in Table 2. For the type *U*_1_, the subscript symbol “*” in $I_{\rho ,*}^{\left(6\right)}$ means that $I_{\rho }^{\left(6\right)}$ is nonzero only when a nonzero second‐order response exists.

Crystal system	Nonvanishing planar components		Nonvanishing currents					Class
	α_*abc*_	β_*abcd*_	*U*_0_ *U*_3_	S_0_ S_1_	U_1_	U_2_ *U*_4_	S_2_	
Isotropic Hexagonal (D_6_, C_6v_, D_6h_) Trigonal(D_3d_, D_3_)	—	*xxyy* = *yyxx* *xyxy* = *yxyx* *xyyx* = *yxxy* *xxxx* = *yyyy* *xxxx* = *xxyy* + *xyxy* + *xyyx*	$I_{\rho }^{\left(1\right)}$ $I_{\rho }^{\left(2\right)}$ $I_{\rho }^{\left(4\right)}$ $I_{\rho }^{\left(5\right)}$ $I_{\rho }^{\left(6\right)}$	$I_{\theta }^{\left(2\right)}$ $I_{\theta }^{\left(3\right)}$ $I_{\theta }^{\left(4\right)}$ $I_{\theta }^{\left(5\right)}$ $I_{\theta }^{\left(6\right)}$	$I_{\rho }^{\left(1\right)}$ $I_{\rho }^{\left(4\right)}$ $I_{\rho ,*}^{\left(6\right)}$	$I_{\rho }^{\left(1\right)}$ $I_{\rho }^{\left(4\right)}$	$I_{\theta }^{\left(2\right)}$ $I_{\theta }^{\left(3\right)}$	E_1_
Trigonal(C_3v_)	*yxx* = *xxy* = *xyx* = − *yyy*							
Cubic (O, T_d_, O_h_) Tetragonal (D_4_,C_4v_, D_4h_, D_2d_)	—	*xxyy* = *yyxx* *xyxy* = *yxyx* *xyyx* = *yxxy* *xxxx* = *yyyy*						
Cubic(T, T_h_)	—	*xxyy*,*yyxx* *xyxy*,*yxyx* *xyyx*,*yxxy* *xxxx* = *yyyy*	all	all	$I_{\rho }^{\left(1\right)}$ $I_{\rho }^{\left(4\right)}$ $I_{\rho }^{\left(5\right)}$ $I_{\rho ,*}^{\left(6\right)}$	$I_{\rho }^{\left(1\right)}$ $I_{\rho }^{\left(4\right)}$ $I_{\rho }^{\left(5\right)}$	$I_{\theta }^{\left(2\right)}$ $I_{\theta }^{\left(3\right)}$ $I_{\theta }^{\left(6\right)}$	E_2_
Monoclinic(C_2h_) Orthorhombic (D_2_,C_2v_,D_2h_)	—	*xxyy*,*yyxx* *xyxy*,*yxyx* *xyyx*,*yxxy* *xxxx*,*yyyy*						
Monoclinic(C_2_, C_1h_)	$\begin{array}{c} xxy,xyx,\\ yxx,yyy \end{array}$							
Hexagonal (C_6_, C_6h_) Trigonal(S_6_)	—	*xxyy* = *yyxx* *xyxy* = *yxyx* *xyyx* = *yxxy* *xxxx* = *yyyy* *yyxy* = −*xxyx* *yxyy* = −*xyxx* *xyyy* = −*yxxx* *xxxy* = −*yyyx* *xxxx* = *xxyy* + *xyxy* + *xyyx* *xxxy* = *yyxy* + *yxyy* + *xyyy*	all	all	$I_{\rho }^{\left(1\right)}$ $I_{\rho }^{\left(2\right)}$ $I_{\rho }^{\left(3\right)}$ $I_{\rho }^{\left(4\right)}$ $I_{\rho }^{\left(6\right)}$	$I_{\rho }^{\left(1\right)}$ $I_{\rho }^{\left(2\right)}$ $I_{\rho }^{\left(3\right)}$ $I_{\rho }^{\left(4\right)}$	$I_{\theta }^{\left(1\right)}$ $I_{\theta }^{\left(2\right)}$ $I_{\theta }^{\left(3\right)}$ $I_{\theta }^{\left(4\right)}$	E_3_
Hexagonal (C_3h_) Trigonal(C_3_)	*xyy* = *yxy* = *yyx* = − *xxx* *yxx* = *xyx* = *xxy* = − *yyy*							
Tetragonal (C_4_, S_4_, C_4h_)	—	*xxyy* = *yyxx* *xyxy* = *yxyx* *xyyx* = *yxxy* *xxxx* = *yyyy* *yyxy* = − *xxyx* *yxyy* = − *xyxx* *xyyy* = − *yxxx* *xxxy* = − *yyyx*						
Triclinic(C_1_,S_2_), other	all	all	all					E_4_

(2) For the starfish‐shaped electrodes in Figure 2b, which are equivalent to the S_0_ type of electrodes in Figure 2h, they collect the azimuthal photocurrent in the region *S* ^θ^(θ_0_,ϕ_0_). For this type of electrodes, the angle ϕ_0_ has to be small enough to confine the auxiliary weight field inside the shadow region; thus, such types of electrodes impose no additional symmetry, and the symmetry cannot be increased simply by changing ϕ_0_. Similar to the U_0_ electrodes, the OAM‐sensitive current *I* ^(1)^ depends on four material parameters: β_*axyy*_ − β_*ayxy*_ and β_*ayxx*_ − β_*axyx*_ with *a = x,y*.

(3) The electrodes can also be designed with other symmetries. The highest symmetry for the U_0_ type of electrodes can be constructed with ϕ_0_ =  2π [45] when the electrodes become annuluses, as illustrated by the U_2_ electrodes shown in Figure 2e. It has circular symmetry, and these coefficients become $G_{1x}^{\rho }=G_{2y}^{\rho }=\pi$ and $G_{1y}^{\rho }=G_{2x}^{\rho }=G_{0x}^{\rho }=G_{0y}^{\rho }=0$. In this case, the currents become:

(14) Iρ1=Imβxxyy−βxyxy+βyyxx−βyxyxP1ϕ02,Iρ2=Reβxxxy+σ2βxyyy−βyxxx−σ2βyyyxP1ϕ02,Iρ3=Reβxyxx−βxxyx−βyxyy+βyyxyP2ϕ02,Iρ4=Imβxxxx+σ2βxyyx+βyxxy+σ2βyyyyP2ϕ02,Iρ5=Reβxxyy+βxyxy−βyxyx−βyyxxP1ϕ02,Iρ6=Imβxxyx+βxyxx+βyxyy+βyyxyP2ϕ02.

It can be seen that each *I* ^(*n*)^ depends on only one independent material parameter formed by the planar response tensor components β, and is independent of α. For example, the OAM‐sensitive current $I_{\theta }^{\left(1\right)}$ is only determined by Im[β_*yxyy*_ − β_*yyxy*_ − β_*xyxx*_ + β_*xxyx*_]. In contrast, for electrodes without additional symmetry, $I_{\theta }^{\left(1\right)}$ is determined by four independent material parameters: Im[β_*xxyy*_ − β_*xyxy*_], Im[β_*xyxx*_ − β_*xxyx*_], Im[β_*yxyy*_ − β_*yyxy*_], and Im[β_*yyxx*_ − β_*yxyx*_]. In addition, we note that similar results in Equation (14) can be achieved in electrodes with lower symmetry, for example, the U_4_ electrodes that collect the photocurrent in the region formed by $\sum_{l=0}^{3}S^{\rho }\left(\frac{l\pi }{2}+\theta_{0},\phi_{0}\right)$ in Figure 2g, which has C_4_ rotational symmetry.

(4) Based on the starfish‐shaped electrodes, we can also design electrodes with C_4_ rotational symmetry, as indicated by the S_2_ electrodes, which collect the photocurrent from the region formed by $\sum_{l=0}^{3}S^{\theta }\left(\frac{l\pi }{2}+\theta_{0},\phi_{0}\;\right)$ in Figure 2j, leading to $G_{0x}^{\theta }=G_{0y}^{\theta }=G_{1x}^{\theta }=G_{2y}^{\theta }=0$ and $G_{1y}^{\theta }=-\;G_{2x}^{\theta }=2\phi_{0}$. The collected azimuthal currents are as follows:

(15) Iθ1=Imβyxyy−βyyxy−βxyxx+βxxyxF12ϕ0,Iθ2=Reβyxxy+σ2βyyyy+βxxxx+σ2βxyyxF12ϕ0,Iθ3=Reβyyxx−βyxyx+βxxyy−βxyxyF22ϕ0,Iθ4=Imβyxxx+σ2βyyyx−βxxxy−σ2βxyyyF22ϕ0,Iθ5=Reβyxyy+βyyxy+βxxyx+βxyxxF12ϕ0,Iθ6=Imβyxyx+βyyxx−βxxyy−βxyxyF22ϕ0.

Similar to Equation (14), here, each $I_{\theta }^{\left(n\right)}$ depends only on one independent material parameter formed by the planar response tensor components β and is independent of α, and the OAM‐sensitive current $I_{\theta }^{\left(1\right)}$ is determined by Im[β_*yxyy*_ − β_*yyxy*_ − β_*xyxx*_ + β_*xxyx*_]. For many crystal symmetries, $I_{\theta }^{\left(1\right)}$ is zero, as shown in Table 3, and this type of electrode is OAM insensitive under the current OAM detection strategy. However, as discussed later in the perspective section, an alternative strategy based on $I_{\theta }^{\left(2\right)}$ can be used for OAM detection in this case.

(5) When the electrodes have inversion symmetry, such as the U_2_, U_4_, and S_2_ electrodes, as well as the U_3_ electrodes, which collect photocurrent from the region formed by $\sum_{l=0}^{1}S^{\rho }\left(l\pi +\theta_{0},\phi_{0}\right)$ and the S_1_ electrodes, which collect photocurrent from the region formed by $\sum_{l=0}^{1}S^{\theta }\left(l\pi +\theta_{0},\phi_{0}\right)$, the inversion symmetry leads to $G_{0x}=G_{0y}=0$, and the photocurrent signals collected from the conventional second‐order photogalvanic effects vanish, regardless of whether the functional material itself possesses inversion symmetry. Therefore, the current from the dipole interaction can be effectively eliminated by a careful electrode design, which provides an effective way to suppress the background signal in OAM direct measurement.

According to the above discussion, the additional symmetry of the electrode geometry can change the dependence of the photocurrent on the response tensor components of α_*abc*_ and β_*abcd*_ by setting different values of the coefficients *F_lja_*: (1) For electrodes with inversion symmetry, the symmetry of the electrodes imposes *F*_0*la*_ =  0, which helps eliminate the conventional second‐order photogalvanic effects. This would save a large category of materials that break inversion symmetry for OAM detection through OPGE because it provides a practical method to reduce the background signal from conventional second‐order photogalvanic effects. (2) When the electrodes possess a symmetry higher than *C*_4_ rotational symmetry, only two of the four quantities *F*_*j*1*x*_, *F*_*j*1*y*_, *F*_*j*2*x*_, and *F*_*j*2*y*_ are nonzero with the same value; thus, all the signals are from the electric‐quadrupole and magnetic‐dipole contributions, and each term of *I* ^(*n*)^ depends only on one independent material parameter, which can be set to zero more easily by choosing a suitable crystal symmetry of the functional material. This approach will be particularly useful in suppressing the background current and increasing the variety of OAM‐sensitive materials for OAM detection.

### 2.3 Crystal Symmetry Analysis for Photocurrents ***I*** ^(***n***)^ with ***n***  =  1, 2, ···, 6

According to the previous discussion, the symmetry of the electrodes has very limited effects on determining whether the values of *I* ^(*n*)^ with *n*  =  1, 2, ···, 6 are zero, but it can effectively suppress the background current from the conventional second‐order response tensor α_*abc*_ by designing electrodes with inversion symmetry and effectively simplify the relationship between each current *I* ^(*n*)^ and the response tensor components of β_*abcd*_, as shown in Equations (9),(14), and (15). For normally incident OAM light, only the planar components of β_*abcd*_ are involved. As a fourth‐rank tensor, all the components of β_*abcd*_ cannot be zero simultaneously, and its nonzero components determine the nonzero terms of *I* ^(*n*)^. When the symmetry of the crystal structure is considered, the dependence of the photocurrents *I* ^(*n*)^ on the response coefficients α_*abc*_ and β_*abcd*_ can be greatly simplified, and the minimal symmetry requirement to provide OAM sensitivity can be obtained by analyzing the Equations (9), (14), and (15) for different crystal systems. With such symmetry analysis, one can obtain knowledge of the class of materials that are OAM sensitive, and the results are listed in Table 3.

In the table, the nonvanishing planar components of α_*abc*_ and β_*abcd*_ are listed, as well as the corresponding nonvanishing current component $I_{\rho /\theta }^{\left(l\right)}$ based on Equations (9), (14), and (15), for different crystal systems. Here, the LG beam is incident perpendicularly to the *a‐b* plane in the coordinates defined by the crystal axes; a similar analysis can be performed for the light incidence along other directions. All the crystal systems are categorized into four classes (E_1_, E_2_, E_3_, and E_4_) according to the nonzero planar components of β_*abcd*_. The E_1_ class includes isotropic, hexagonal (D_6_, C_6v_, D_6h_), trigonal (D_3d_, D_3_, C_3v_), cubic (O, T_d_, O_h_), and tetragonal (D_4_, C_4v_, D_4h_, D_2d_) crystals. In the E_1_ class, only $I_{\rho }^{\left(1\right)}$, $I_{\rho }^{\left(4\right)}$, $I_{\theta }^{\left(2\right)}$, and $I_{\theta }^{\left(3\right)}$ can be nonzero for the electrodes U_2_, U_4_, and S_2_ with the highest symmetry; the radial signal is $I\;\left(m,\sigma \right)=m\sigma_{i}I_{\rho }^{\left(1\right)}+I_{\rho }^{\left(4\right)}$, and the azimuthal signal is $I\;\left(m,\sigma \right)=mI_{\theta }^{\left(2\right)}+\sigma_{i}I_{\theta }^{\left(3\right)}$. For *U*_1_ electrodes, the radial signal is $I\;\left(m,\sigma \right)=m\sigma_{i}I_{\rho }^{\left(1\right)}+I_{\rho }^{\left(4\right)}+\sigma_{r}I_{\rho }^{\left(6\right)}$, but $I_{\rho }^{\left(6\right)}$ arises from the conventional photogalvanic effects only. For the electrodes *U*_0_, *U*_3_, *S*_0_, and *S*_1_, the symmetry is much lower, and more terms of *I* ^(*n*)^ become nonzero, but $I_{\theta }^{\left(1\right)}$ is still zero. Therefore, for the existing detection strategy based on CPGE current extraction, only the radial current (*I* ^(1)^) can be collected with the background CPGE current *I* ^(3)^ =  0, but no OPGE current can be obtained from the azimuthal current. However, because the term $mI_{\theta }^{\left(2\right)}$ is linearly related to the OAM order *m*, in principle, OAM detection is also possible if the term $mI_{\theta }^{\left(2\right)}$ can be extracted from the azimuthal current when adopting appropriate method, as discussed in the last section. The E_2_ class includes cubic (T, T_h_), monoclinic (C_2h_, C_2_, C_1h_), and orthorhombic (D_2_, C_2v_, D_2h_) crystals. For electrodes U_0_, U_3_, S_0_, and S_1_ with lower symmetry, all terms of I^(n)^ are nonzero. While for electrodes with higher symmetries, compared to the E_1_ class materials, the E_2_ class materials have two additional nonzero photocurrent response terms from $m\sigma_{r}I_{\rho }^{\left(5\right)}$ and $\sigma_{r}I_{\theta }^{\left(6\right)}$. For the existing detection strategy based on CPGE current extraction, both the $m\sigma_{r}I_{\rho }^{\left(5\right)}$ and $\sigma_{r}I_{\theta }^{\left(6\right)}$ terms do not contribute to the CPGE because the incident OAM beam is circularly polarized with σ_*r*_ =  0. The E_3_ class includes hexagonal (C_6_, C_6h_, and C_3h_), trigonal (S_6_ and C_3_), and tetragonal (C_4_, S_4_, and C_4h_) crystals. For the E_3_ class, the photocurrent response becomes *I*(*m*, σ) =  *m*σ_*i*_ *I* ^(1)^ + *mI* ^(2)^ + σ_*i*_ *I* ^(3)^ + *I* ^(4)^ for both the radial current and azimuthal current under circularly polarized LG beams. All current components exist for materials in the E_3_ class, and the response has to be analyzed case by case. While for the E_4_ class, which includes all the materials that are not categorized into the previous three classes, all the components of *I* ^(*n*)^ for *n*  =  1, 2, ···, 6 are nonzero.

Therefore, from a symmetry point of view, after combining the electrodes’ symmetry and the crystal symmetry, all materials are OAM sensitive in the radial currents in the conventional OPGE detection scheme; those materials from classes E_3_ and E_4_ are OAM sensitive in the azimuthal currents for electrodes with higher symmetries; and materials from the E_2_ classes can be OAM sensitive in the azimuthal currents for electrodes with much lower symmetries.

### 2.4 Experimental Progress

**FIGURE 3:**
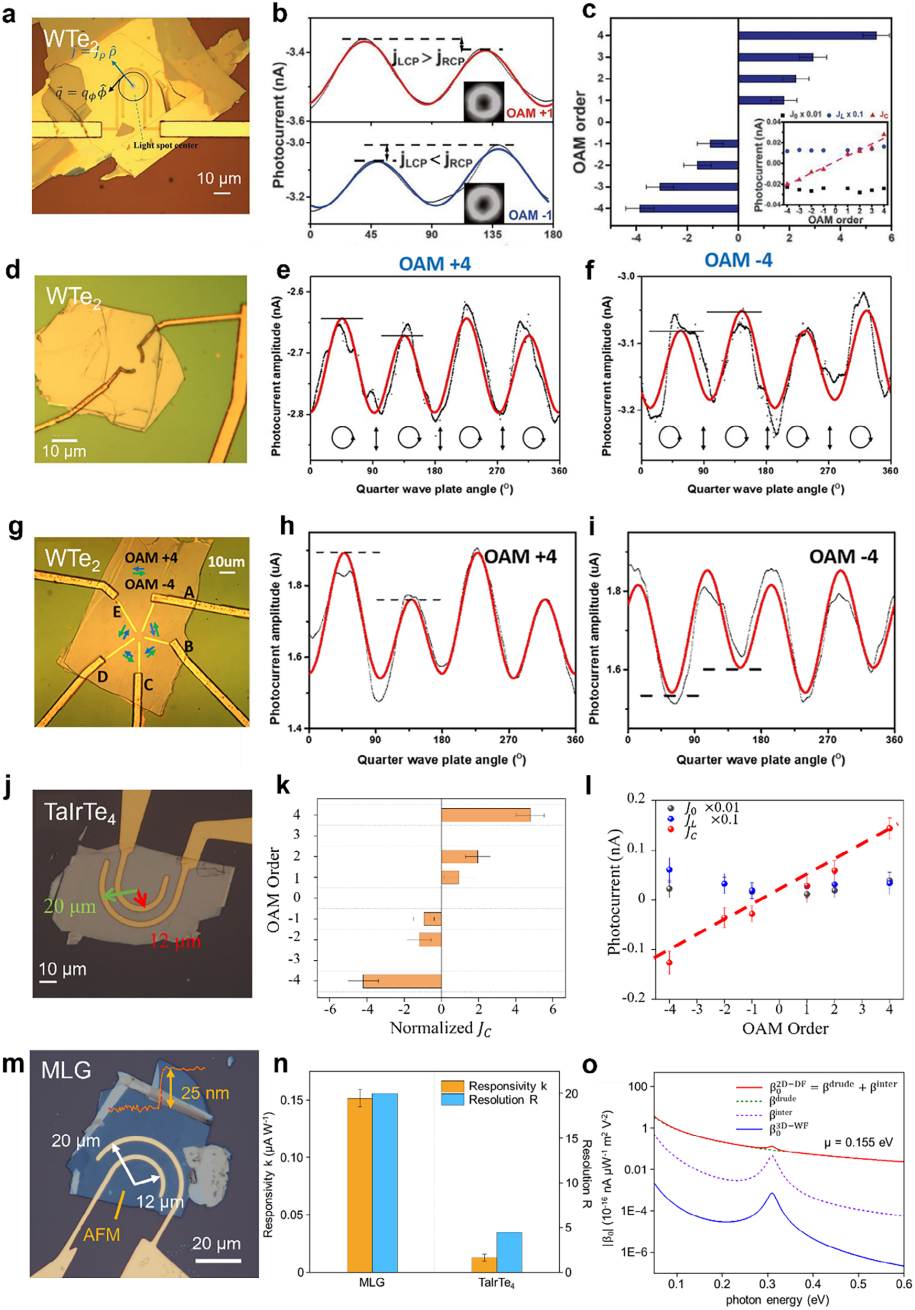
Experimental results and progress of OPGE‐based OAM photodetection. (a–c) Experimental results of the U‐shaped WTe_2_ device, including an optical image (a), the measured photocurrent response under the excitation of OAM ±1 beams as a function of the QWP angle (b), and the normalized CPGE response as a function of the OAM order ranging from −4 to +4 (c). (a–i) Reproduced with permission [32], Copyright 2020, American Association for the Advancement of Science. (d–i) Experimental results of Ω‐shaped (d–f) and starfish‐shaped (g–i) WTe_2_ devices, including optical images (d, g) and CPGE measurements for OAM orders +4 (e, h) and ‐4 (f, i). (j–l) Reproduced with permission [40], Copyright 2022, John Wiley & Sons. Experimental results of the U‐shaped TaIrTe_4_ device, including an optical image (j), normalized CPGE response as a function of the OAM order ranging from −4 to +4 (h), and linear fitting of the CPGE, anisotropic, and polarization‐independent components of the measured photocurrent as a function of the OAM order (l). (m‐o) Reproduced under the terms of the CC‐BY Creative Commons Attribution 4.0 International license (https://creativecommons.org/licenses/by/4.0) [42]. Copyright 2025, The Authors, published by Springer Nature. Experimental results and analysis of the U‐shaped multilayer graphene device, including optical images (m), comparison of the OPGE response and OAM resolution capability with those of the U‐shaped TaIrTe_4_ device (n), and calculations of the OPGE response coefficients for 2D‐dirac fermions and 3D‐weyl fermions (o).

Based on the above symmetry analysis, a device made from photosensitive materials with an OPGE response and correct electrode geometry should be able to resolve the OAM order when the circular polarization‐dependent photocurrent is extracted from a standard CPGE measurement. The pioneering work was performed by Ji et al., using WTe_2_. The typical device is based on a U‐shaped electrode geometry (Figure 3a), which can effectively collect the radial photocurrent response when the OAM beam is embedded between the inner and outer electrodes. To extract the CPGE response, a quarter‐wave plate after a linear polarizer is employed and rotated to modulate the polarization of the incident OAM beam. The polarization of the OAM beam undergoes a periodic change of linear (0°)–left circular (45°)–linear (90°)–right circular (135°)–linear (180°) polarization states in an operation cycle of 180°. By measuring the photocurrent with a certain degree interval, the CPGE response can be directly extracted via Fourier transform, which corresponds to the 180°‐periodicity component of the photocurrent (Figure 3b). The experimental results show that the extracted CPGE response displays a linear and steplike dependence on the topological charge of the OAM beam, which enables the resolution of the OAM order to range from +4–4 (Figure 3c). However, the linear polarization‐dependent current *J*_L_ (90°‐periodicity component) and the polarization‐independent thermal current *J*_0_ do not have such dependence on the OAM order.

Since WTe_2_ has an OPGE response in both the radial and azimuthal directions, three different electrode geometries are tested, and all the results clearly demonstrate a CPGE response that reverses in sign when the OAM number goes opposite (Figure 3d–i). However, U‐shaped electrode geometries usually demonstrate better performance than other electrode geometries because this geometry is more tolerant of position errors at different OAM orders than the starfish‐electrode device is. The OPGE detector based on WTe_2_ works in the near‐infrared region but has a very limited response in the mid‐infrared region. Later on, Lai et al. replaced WTe_2_ with TaIrTe_4_, a type‐II Weyl semimetal with exactly the same C_2v_ crystal symmetry as WTe_2_, realizing OAM detection in the mid‐infrared region on the basis of the same device geometry (Figure 3j–l). This success mainly takes advantage of the topologically enhanced responsivity at the mid‐infrared region while maintaining the OPGE response that is allowed by C_2v_ symmetry. In a more recent work by Yang et al., mid‐infrared OAM detection was also realized with multiple‐layer graphene (MLG) with unexpectedly high OAM resolution compared with TaIrTe_4_ (Figure 3m,n). Compared with TaIrTe_4_, the absolute responsivity in the mid‐infrared region underscores the advantage of MLG in photoelectric conversion. The improved response is due to two reasons: 1. the crystal symmetry D_6h_ of the MLG only allows OAM sensitive photocurrent collection with U‐shaped electrodes, but the CPGE background signal is suppressed with this collection geometry, which suppresses noise from the background; 2. the response coefficients are greatly enhanced due to the low‐dimensional effect, which is induced mainly by Drude‐type intraband resonant transitions for massless Dirac Fermions. Such dimensionality enhancement only occurs in two dimensions instead of three dimensions, as discussed in detail in the work [42] (Figure 3o). Because MLG is an epitaxially growable material with high stability in moderate environments and can be easily integrated with the silicon chip technique [52, 53, 54, 55, 56], the successful demonstration of an MLG‐based OAM detector paves the way for the development of on‐chip detectors and large‐scale FPA devices (Figure 1a,b).

## 3 Operation Speed

**FIGURE 4:**
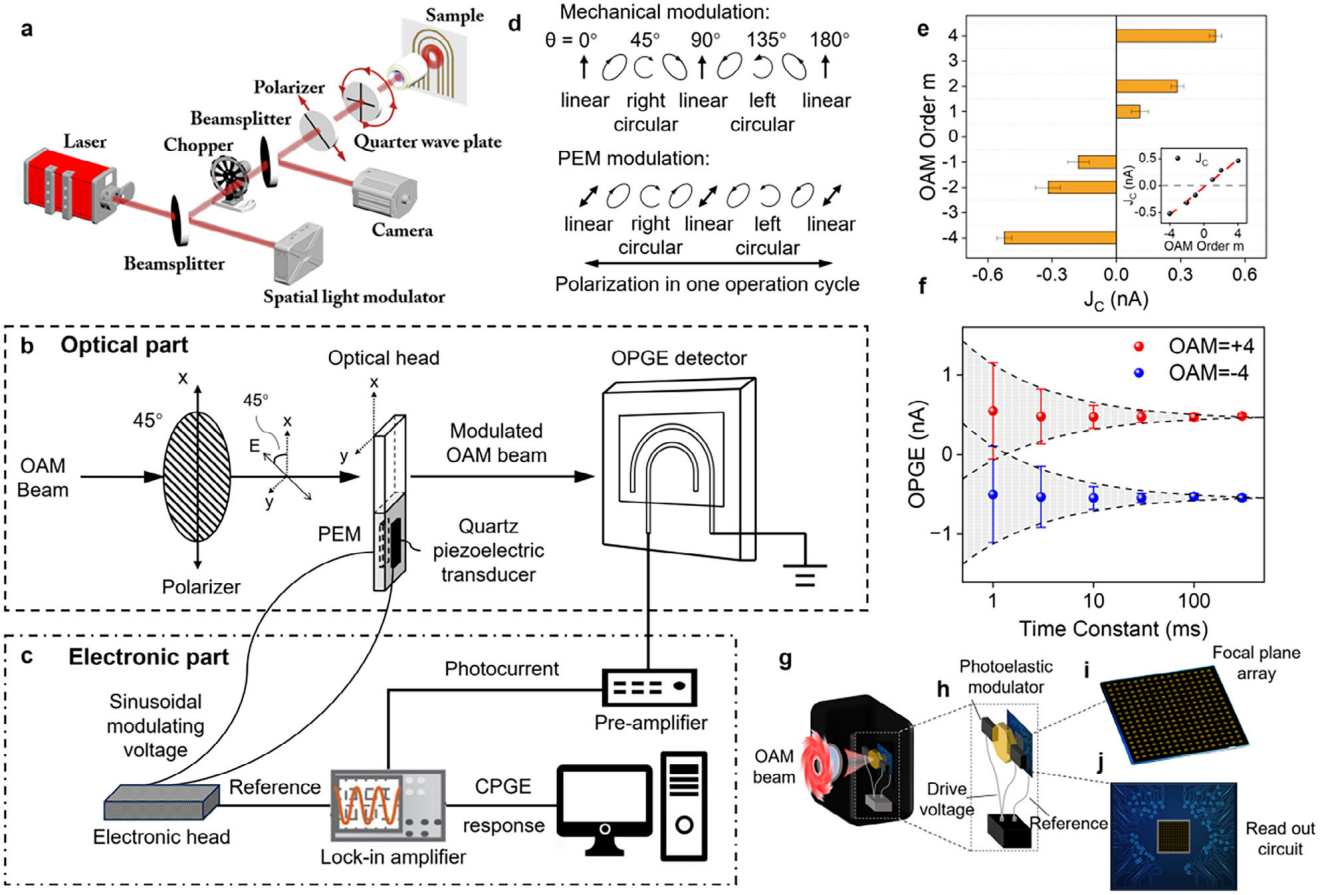
Schematic of PEM modulation and high‐speed OAM photodetection experiments. (a) Experimental setup for OPGE measurement based on mechanical modulation. Reproduced with permission [32], Copyright 2020, American Association for the Advancement of Science. (b,c) Experimental setup including an optical part (b), polarization modulation with the polarizer and optical head of the PEM, and an electronic part (c), modulating voltage, photocurrent collection, and CPGE extraction) for OPGE measurements based on PEM modulation [41]. (d) Comparison of the polarization variation in one operation cycle between mechanical and PEM modulations [41]. (e) Measured CPGE response J_C_ based on PEM modulation, together with its linear fit (inset), as a function of the OAM order m [41]. (f) Measured CPGE response together with its uncertainty as a function of the time constant of the lock‐in amplifier under the excitation of OAM beams with an OAM order of ±4 [41]. (g–j) Schematic of the application of the PEM modulation scheme for light OAM photodetector focal‐plane‐array devices based on graphene, including the overall device structure of the focal‐plane‐array device (g), schematic of the OAM detection chip and PEM modulation module driven by the power module (h), the focal‐plane array based on MLG photodetectors (i), and the read‐out circuit (j) [41]. (b–j) Reproduced with permission [41], Copyright 2025, SPIE.

In a typical OPGE response measurement, the circular polarization‐dependent component of the photocurrent response must be extracted to distinguish the OAM orders of light. Simultaneous measurement of the left‐ and right‐circular polarization‐dependent photocurrents was realized through continuous rotation of a quarter waveplate (QWP) in early works [32, 40, 42], as illustrated in Figure 4a. The CPGE component is then extracted via Fourier transform of the QWP angle‐dependent photocurrent response. Limited by the speed of mechanical polarization modulation, the operation speed is at the minute level for early demonstration, which cannot fulfill the speed requirements of most applications [32, 40, 42]. The slow operation speed is a major drawback of OPGE‐based OAM detectors compared with parallel OAM detection technology based on SPPs, which can reach operation speeds on the order of tens of microseconds [34]. In a recent work by Yang et al., the operation speed was greatly increased to the millisecond level via an electrical polarization modulation strategy with a photoelastic modulator (PEM) accompanied by a phase‐locked readout approach with a lock‐in amplifier. A detailed schematic diagram of their modulation and measurement scheme is shown in Figure 4b,c. The PEM used in this work consists of a ZnSe_2_ resonant bar operating at a resonance frequency of 50.14 kHz, which is capable of mid‐infrared circulation polarization modulation. When the OAM beam passes through the PEM, in a single operational cycle of the PEM, the polarization of the OAM beam undergoes a sequence of transitions—linear, left‐circular, linear, right‐circular, linear, exhibiting a variation pattern of polarization modulation similar to that realized by rotating the QWP over a period of 180° (Figure 4d,f). In the detection part, with an OPGE‐based OAM detector made from multilayer graphene (MLG), the topological charge of light OAM can be clearly distinguished by the quantized plateau of the CPGE response, which is directly extracted by a lock‐in amplifier that is phase locked to the modulation signal of the PEM (Figure 4c). In this work, the readout speed is mainly limited by the 300 ms measurement time constant of the lock‐in amplifier (Figure 4g). The measurement time constant of the lock‐in amplifier can be reduced to compensate for the elevated noise level, and its signal‐to‐noise level barely allows a measurement time constant of 1 ms (Figure 4h). Providing enough OPGE response of the device, the measurement time of the lock‐in can be further decreased, and the operation speed could be improved further under this modulation scheme. The time constant is ultimately limited by the polarization modulation frequency of the PEM and the photocurrent response time of the multilayer graphene device, both of which could be improved further in the future [57, 58, 59]. In addition, the electric modulation scheme and lock‐in readout scheme are directly applicable to focal plane array devices and on‐chip integration (Figure 4i,j). For the focal plane array device, the modulation crystal with the transducer assembly can be placed before the OAM detector arrays, and the phase‐sensitive reading circuit can be integrated with the photocurrent readout circuit and locked to the electric driving source to extract the CPGE component of each detector cell. For on‐chip integration, miniaturized electrically driven polarization modulation materials are required for OAM detectors, and this is possible with atomically thin topological semimetals for tunable phase retardation [60].

## 4 Challenges and Perspectives

Experimentally demonstrated direct OAM devices are all based on CPGE measurements, and the extraction of the OPGE current requires the modulation of the polarization states of LG beams, which leads to a complicated detection procedure. In fact, on the basis of our symmetry analysis shown in Table 3, various detection schemes could make the detection procedure simpler. For example, when a highly symmetric electrode S_2_ is used for materials with E_1_ class symmetry, direct detection based on the photocurrent response *I* ^dc^(*m*,σ) = m*I* ^(2)^ + σ_*i*_ *I* ^(3)^ can be performed, and the detected current can be directly quantified with the OAM order *m* as long as the LG beam is linearly polarized or unpolarized; this direct extraction strategy can greatly improve the detection speed, which is limited by the polarization modulation and related extraction process in the conventional detection scheme. However, experimentally, such a detection scheme may suffer from a photocurrent response that results from trivial effects that are OAM dependent. For example, different beam profiles at different OAM orders can contribute to an OAM‐dependent photocurrent response and interfere with detection. Furthermore, detection can also be interfered with by other photocurrent response mechanisms that are OAM sensitive, such as the s‐PGE response reported in previous work [61]. In light of these difficulties, more experimental and theoretical studies are expected to explore this new detection scheme.

**FIGURE 5:**
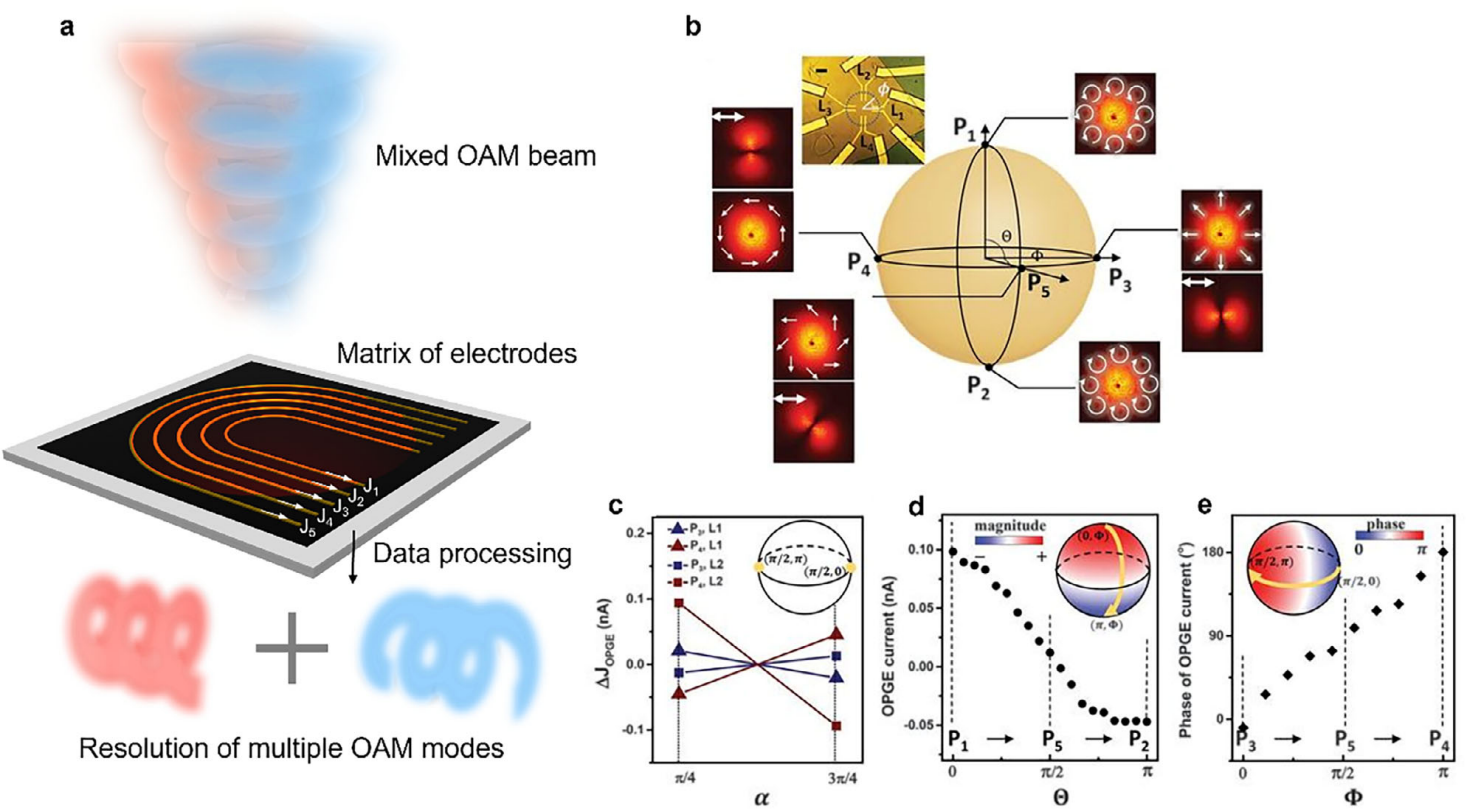
Prospects for detecting more complex vortex beams. (a) Schematic of the device geometry and process for measuring arbitrary mixtures of OAM beams. (b–e) Reproduced with permission [32], Copyright 2020, American Association for the Advancement of Science.Experimental example and prospects for direct detection of a vectorial OAM beam represented on a higher‐order Poincaré sphere (HOPS), including the following: (b) Schematic of *m* = 1, σ = −1 HOPS, with states represented by (Θ, Φ) spherical coordinates and five points P_1_ to P_5_ together with their polarization distributions with or without a linear polarizer oriented in the horizontal direction and their corresponding intensity profiles. (Inset) Optical image of the octopus‐shaped electrodes with four pairs of electrodes (L_1_, L_2_, L_3_, and L_4_) located at four azimuthal coordinates (ϕ = 0, π/2, π, and 3π/2, respectively). (c) Relative photocurrent amplitudes at two QWP angles (π/4 and 3π/4) from two states (P_3_ and P_4_) on HOPS measured at two locations (L_1_ and L_2_). (d) OPGE current amplitude from a set of states on the line connecting P_1_, P_5_, and P_2_ with the same Φ, and (e) phase of the OPGE current from a set of states on the line connecting P_3_, P_5_, and P_4_ with a fixed Θ.

To date, all discussions are limited to the detection of single scalar OAM beams, and there is a direct correspondence between the OPGE current and the topological charge of OAM beams, which enables direct detection of scalar OAM beams. A step further involves the detection of a mixture of different OAM orders, and a protocol for detecting such a mixture was proposed in the first OPGE detector by Ji et al. [32], who suggested the use of a well‐designed matrix of electrodes (Figure 5a) for this purpose. Considering the mixture of *n* LG modes $E(LG_{0}^{1})$… $E(LG_{0}^{n})$ with percentages *x*_1_…*x*_n_, the mixed light field and OPGE photocurrent intensities are expressed as:

(16) $$\begin{array}{ccc} E{\left(\rho ,\theta \right)}_{mix} & = & x_{1}E\left(LG_{0}^{1}\right)+x_{2}E\left(LG_{0}^{2}\right)+\cdots +x_{n}E\left(LG_{0}^{n}\right)J_{OPGE,mix}\\ & & \propto \frac{e^{-2\rho^{2}/w_{0}^{2}}}{\rho }\left(\frac{x_{1}}{\left|1\right|!}\rho^{2}+\frac{2x_{2}}{\left|2\right|!}\rho^{4}+\cdots +\frac{nx_{n}}{\left|n\right|!}\rho^{2n}\right) \end{array}$$

where ρ is the radial coordinate. As the intensity profiles of different OAM mixture is different, the detection with a series of electrodes designed with different radii can, in principle, distinguish the mixture of modes when accompanied by proper post‐data processing.

An even more complicated case involves vectorial OAM beams, which process space‐variant states of polarization in addition to the helical phase distribution. This was also discussed in the very first OPGE detector work by Ji et al., [32]. Vectorial OAM beams can be represented on a higher‐order Poincaré sphere (HOPS) [62, 63] (Figure 5b). In the parameter space (represented by the spherical coordinates Θ and Φ) of the HOPS, the state of the optical field is represented by $\left|\Psi \left(\Theta ,\Phi \right)\right\rangle =\cos\left(\frac{\Theta }{2}\right)\exp\left(-\frac{i\Phi }{2}\right)|L_{-m}\rangle +\sin\left(\frac{\Theta }{2}\right)\exp\left(\frac{i\Phi }{2}\right)\left|R_{m}\right\rangle$, where |*R_m_* 〉 and |*L*_−*m*_〉 are scaler vortex beams with OAM +m (−m) and SAM σ = −1 (+1), respectively. The OPGE response can be divided into two parts, $J_{\phi ,OPGE}^{\left(0\right)}$ and $J_{\phi ,OPGE}^{\left(\phi \right)}$, according to their dependence on the azimuthal angle ϕ, expressed as:

(17) $$\begin{array}{ccc} J_{\phi ,OPGE} & = & J_{\phi ,OPGE}^{\left(0\right)}+J_{\phi ,OPGE}^{\left(\phi \right)}\#\propto \frac{m}{\rho }\left[\left(c_{0}+c_{1}\cos\Theta \right)\right.\\ & & \left.+\left(c_{2}\cos\left(2\left(m+\sigma \right)\phi +\Phi \right)\right.\right.\\ & & +\left.\left.c_{3}\sin\left(2\left(m+\sigma \right)\phi +\Phi \right))\sin\left(\Theta \right)\right)\right] \end{array}$$

where *c*_0_, *c*_1_, *c*_2_, and *c*_3_ are conductivity coefficients. To capture this azimuthal angle dependence, the electrodes were arranged in an “octopus” shape (Figure 5b, inset) to enable a set of azimuthal current measurements at various azimuthal coordinates (L_1_–L_4_), while the beam was fixed at the center defined by the electrodes. The experimental results show that for both the P_3_ (π/2, 0) and P_4_ (π/2, π) states, the difference in the OPGE response at two QWP angles (π/4 and 3π/4) and $\Delta J_{OPGE}^{\left(\phi \right)}$ collected at different azimuthal angles L_1_ and L_2_ are of opposite sign, indicating the existence of an $J_{OPGE}^{\left(\phi \right)}$ that originates from the vectorial OAM beams (Figure 5c). Moreover, the experimental results show that the amplitude of the OPGE current directly corresponds to Θ when Φ is fixed, and the phase of the OPGE current can be mapped onto the Φ coordinate on HOPS when Θ is fixed (π/2) (Figure 5d–e), indicating the distinct OPGE response related to the states on the HOPS. On the basis of these results, it can be expected that the OAM order or the coordinates of any arbitrary OAM state on a HOPS can be specifically determined by measuring currents via a small matrix of electrodes with suitable data processing.

With the successful demonstration of these single devices, an important step forward is to integrate them into a chip or FPA devices, which is evitable for practical application [64] such as OAM imaging and multi‐dimensional light detection. However, it would be very challenging to design an accurate OAM detector for imaging purposes as well as to develop an efficient method to extract the OAM order. This is because the detection of OAM order in each pixel requires the global information of the light, and the detection accuracy strongly depends on the relative position between the OAM light and the collection electrodes. The challenge can be solved by resolving full optical parameters using a deep learning network, which has been successfully demonstrated in recently developed multifunctional photodetectors [65, 66]. By integrating the OAM detector, intensity detector [67, 68], and polarization detector [69] as a unit of an FPA device, the existing non‐local high‐dimensional photodetector [65] can be further expanded to add OAM sensitivity.

The successful demonstration of graphene for OAM detection with high recognition capability is promising for large‐scale FPA device integration. As the properties of graphene are versatilely tunable by an external gate [64], it facilitates intelligent detection through a deep learning network [66]. Following the previous successful demonstration of full‐Stokes parameters and wavelength detection through a twisted double bilayer graphene photodetector with a trained convolutional neural network [64], it will be possible to detect the OAM order, intensity, and polarization in one detector using twisted bilayer graphene as a functional material and further scale it up as an FPA working unit in the midinfrared region [64]. Regardless of the scheme adopted, the extension of a single OAM detector to an FPA device is evitable for practical applications [64]. Furthermore, although graphene seems to be a strong candidate from the consideration of many aspects, our symmetry analysis of sensitive materials and related electrode designs will provide more space for the discovery and development of more high‐quality materials. In addition, the intrinsic quantum geometries of various quantum materials present many opportunities for OAM detection via OPGE, together with its integration for full optical parameter detection and large‐scale on‐chip integration.

## Author Contributions

Jinluo Cheng contributed equally to the conceptualization, funding acquisition, project administration, supervision, and writing (original draft, review and editing) of the study, and took a leading role in methodology development and validation. Dehong Yang contributed equally to visualization and provided supporting contributions to the writing of the original draft and to review and editing. Weiming Wang provided supporting contributions to methodology and writing of the original draft and contributed equally to visualization. Chang Xu contributed to the writing of the original draft and to reviewing and editing in a supporting role. Zipu Fan contributed equally to visualization and provided supporting contributions to the writing of the original draft and to review and editing. Dong Sun contributed equally to the conceptualization, data curation, funding acquisition, project administration, supervision, and writing (original draft, review and editing) of the study.

## Conflicts of Interest

The authors have no conflicts to disclose.

## Data Availability

Data sharing is not applicable to this article as no new data were created or analyzed in this study.
